# Mechanistic insight into the suppression of microglial ROS production by voltage-gated proton channels (VSOP/Hv1)

**DOI:** 10.1080/19336950.2017.1385684

**Published:** 2017-11-17

**Authors:** Takafumi Kawai, Shoki Tatsumi, Shinji Kihara, Kenji Sakimura, Yasushi Okamura

**Affiliations:** a Integrative Physiology, Department of Physiology, Graduate School of Medicine, Osaka University, Osaka, JAPAN; b Graduate School of Frontier Biosciences, Osaka University, Osaka, JAPAN; c Department of Biomedical Informatics, Division of Health Sciences, Graduate School of Medicine, Osaka University, Osaka, JAPAN; d Department of Cellular Neurobiology, Brain Research Institute, Niigata University, Niigata, JAPAN

**Keywords:** microglia, microglial activation, ROS, voltage-gated proton channel, VSOP/Hv1

## Abstract

Voltage-gated proton channels (VSOP/Hv1) reportedly promote reactive oxygen species (ROS) production in several immune cell types. However, we recently reported that primary microglia from VSOP/Hv1-deficient mice show higher ROS production than those from WT mice. Microglia may show a distinct activation status between WT and VSOP/Hv1-deficient cells, leading to a distinct level of ROS production between them. This is unlikely, however, because ROS production in VSOP/Hv1-deficient microglia remained higher than in WT microglia when the cells were exposed to LPS. Further, this increase in ROS production in VSOP/Hv1-deficient cells was not observed in macrophages, which suggests microglia have a unique mechanism of VSOP/Hv1-dependent ROS regulation. The mechanism underlying this unconventional ROS regulation by VSOP/Hv1 in microglia is discussed.

## Introduction

VSOP/Hv1, which is encoded by the gene *Hvcn1*, is a voltage gated proton channel consisting of a voltage-sensor domain without the pore domain characteristic of other ion channels.^1,2^ The voltage-sensor domain plays a dual role as both a proton permeation pathway and voltage sensor. VSOP/Hv1 has diverse biological functions, but its role in the regulation of reactive oxygen species (ROS) production in various immune cell types, including neutrophils, eosinophils, B lymphocytes, T lymphocytes, macrophages and microglia,^3-12^ has been the most intensively studied. VSOP/Hv1 is thought to support ROS production in these cells by exporting excess intracellular protons that accumulate during respiratory bursts, as intracellular acidification and membrane depolarization inhibit nicotinamide adenine dinucleotide phosphate (NADPH) oxidase activity.^6,13^ On the other hand, we recently reported that extracellular ROS production is markedly higher in primary cultures of *Hncn1*^−/−^ microglia than in WT microglia, which suggests VSOP/Hv1 also exerts suppressive effects on ROS production in microglia.^14^ Moreover, our findings indicated that the impact of VSOP/Hv1-mediated ROS regulation on neuronal damage during brain ischemia appears to depend on the age of the animals, indicating that the effect of VSOP/Hv1 on microglial ROS levels is dependent on the state of the microglia. Considering the potential significance of VSOP/Hv1 as a therapeutic target for ischemic stroke or other neuropathology,^12,14,15^ it is important to fully understand the mechanisms by which VSOP/Hv1 affects ROS production. In the present study, we performed several experiments to gain insight into the mechanism of the suppressive ROS regulation by VSOP/Hv1.

## Results

### Analysis of gp91 expression and ROS production in WT and Hvcn1^−/−^ microglia with and without LPS treatment

**Figure 1.**
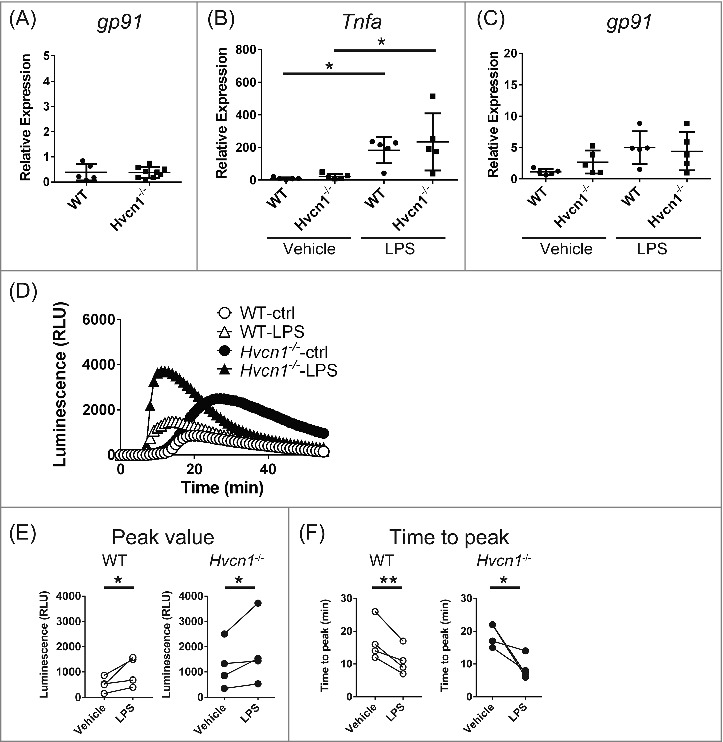
Comparison of gene expression and ROS production between WT and *Hvcn1*^−/−^ microglia in LPS-treated or untreated groups. (A) Using quantitative PCR, *gp91* mRNA levels were compared between WT and *Hvcn1*^−/−^ microglia. There was no obvious difference between them (P = 0.9308, unpaired t-test, n = 5 for WT, n = 9 for *Hvcn1*^−/−^). (B, C) *Tnfa* and *gp91* mRNA levels in WT and *Hvcn1*^−/−^ microglia with or without LPS treatment for 2 days. (B) Both WT and *Hvcn1*^−/−^ microglia showed obvious increases in *Tnfa* expression, confirming the inflammatory responses in these cells. There was no significant difference in *Tnfa* expression between WT and *Hvcn1*^−/−^ microglia in either vehicle or LPS treatment groups (P > 0.9999). (C) LPS treatment moderately upregulated *gp91* expression (in WT, P = 0.0564), but there was no difference between WT and *Hvcn1*^−/−^ microglia in either the vehicle or LPS treatment groups (P > 0.9999). *P < 0.05, Bonferroni multiple comparisons, multiplicity adjusted P-values, n = 5 for each. (D) Representative chemiluminescence signals showing the time course of extracellular superoxide anion production by WT or *Hvcn1*^−/−^ microglia in vehicle and LPS treatment groups; 1 μM PMA was applied at 5 min. LPS treatment obviously accelerated the response and increased its magnitude in both WT and *Hvcn1*^−/−^ microglia. ROS production was enhanced in *Hvcn1*^−/−^ microglia. (E, F) Statistical data showing that LPS increases and accelerates ROS production in WT and *Hvcn1*^−/−^ microglia. (E) Peak values of PMA responses are significantly upregulated by LPS in both WT and *Hvcn1*^−/−^ microglia. *P<0.05, ratio paired t-test. (F) Time to peak of the PMA response is significantly reduced by LPS both in WT and *Hvcn1*^−/−^ microglia. *P<0.05 and **p<0.01, ratio paired t-test.

In microglia, the NADPH oxidase complex plays a crucial role in respiratory bursts.^16-19^ There are several possible mechanisms that could account for the upregulated ROS generation in *Hvcn1^−/−^* microglia. For example, because the assembly of NADPH oxidase from membrane-bound and cytosolic subunits is essential for enzyme function, ROS production could be enhanced by promoting translocation of cytosolic subunits to the membrane or by phosphorylating subunits.^17-19^ We recently reported that altered actin dynamics, which governs trafficking of the subunits to the membrane,^20-23^ accounts at least in part for the increased ROS production in *Hvcn1^−/−^* microglia.^14^ Nevertheless, it is likely that factors other than actin dynamics also contribute to the enhanced ROS production in *Hvcn1^−/−^* microglia. Specifically, the expression of NADPH oxidase subunits is frequently upregulated upon activation of microglia,^24,25^ which could contribute to the enhanced ROS production seen in *Hvcn1^−/−^* microglia. We therefore compared levels of *gp91* mRNA, which encodes the transmembrane component of NADPH oxidase, between WT and *Hvcn1^−/−^* microglia. However, we found that there was no significant difference in *gp91* expression between WT and *Hvcn1^−/−^* microglia (Fig. 1A; P = 0.9308, unpaired t-test). We then examined the effect of microglial activation on *gp91* expression and ROS production by treating WT and *Hvcn1^−/−^* microglia with 100 ng/ml LPS for 2 days. The pro-inflammatory response of microglia was observed in both genotypes, as evidenced by the significant upregulation of *Tnfa* (Fig. 1B; P < 0.05 for both, Bonferroni multiple comparisons, multiplicity adjusted P-values). On the other hand, we saw no difference in the levels of *Tnfa* expression between WT and *Hvcn1^−/−^* microglia in either the vehicle or LPS treatment groups (Fig. 1B; P>0.9999 for both, Bonferroni multiple comparisons, multiplicity adjusted P-values). We also analyzed *gp91* expression in the same groups used to assess *Tnfa* expression. Consistent with an earlier report that inflammatory responses upregulate *gp91* expression^24,25^, cells treated with LPS tended to show greater *gp91* expression than vehicle-treated groups (Fig. 1C; in WT, P = 0.0564, Bonferroni multiple comparisons, multiplicity adjusted P-value). However, we detected no difference in *gp91* expression between WT and *Hvcn1^−/−^* microglia in the LPS treatment group (Fig. 1C; P>0.9999, Bonferroni multiple comparisons, multiplicity adjusted P-values). When we then measured extracellular ROS induced by 1 μM PMA in the LPS and vehicle treatment groups (Fig. 1D), we consistently observed that LPS accelerates and strengthens the ROS production in both WT and *Hvcn1^−/−^* microglia, as evidenced by the increase in peak amplitude (Fig. 1E) and decrease in the time to peak (Fig. 1F). Notably, we also found that ROS production by *Hvcn1^−/−^* microglia remains higher than the production by WT microglia in the LPS treatment groups (Fig. 1D, E).

### Enhanced ROS production in the absence of VSOP/Hv1 is observed in microglia but not in macrophages

**Figure 2.**
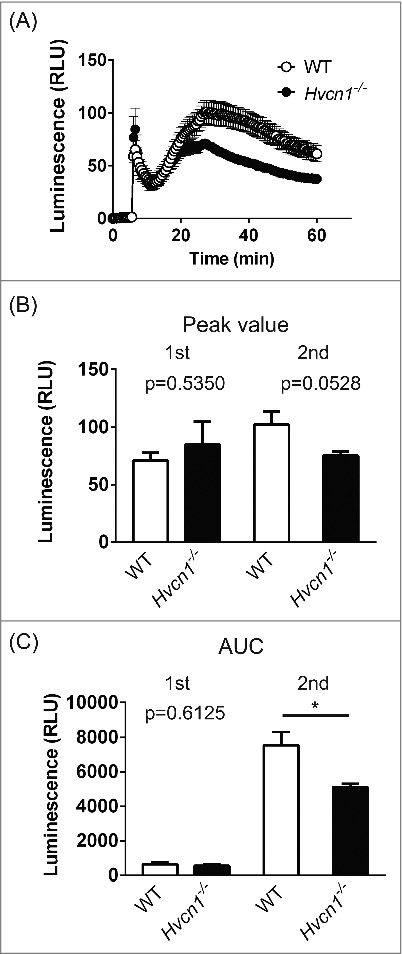
Comparison of ROS production in WT and *Hvcn1*^−/−^ peritoneal macrophages. (A) Time course of extracellular superoxide anion production in WT (open circles) and *Hvcn1*^−/−^ (filled circles) macrophages after stimulation with 1 μM PMA. After PMA was applied at 5 min, bimodal responses were observed. Data are means ± SEM. P<0.05, two-way ANOVA, n = 5 for each. (B) Luminescence at the first and second peaks in WT and *Hvcn1*^−/−^ macrophages. There was a moderate difference at the second peak (P = 0.0528; unpaired t-test). (C) Area under the response curve (AUC) calculated for first and second peaks. For the first peak, the calculation goes from the onset to the valley in the bimodal responses. For second peak, the calculation goes from the valley to the end of recording. At the second peak, there was a significant difference between WT and *Hvcn1^−/−^* macrophages. *P<0.05, unpaired t-test.

Because we observed enhanced ROS production in *Hvcn1^−/−^* microglia, irrespective of their activation status, we examined whether a similar phenomenon also occurs in *Hvcn1^−/−^* macrophages, which share multiple features in common with microglia. In contrast to microglia, *Hvcn1^−/−^* macrophages showed weaker ROS production in response to PMA than did WT macrophages (Fig. 2A, P<0.01, two-way ANOVA, n = 5 for each). When peritoneal macrophages were stimulated with PMA, bimodal responses were observed both in WT and *Hvcn1^−/−^* cells that had also been observed in neutrophils and T-cells.^8,14^ At the second peak, the response of *Hvcn1^−/−^* macrophages was about 30% smaller than in WT macrophages (Fig. 2B and C, in WT vs *Hvcn1^−/−^*: for peak value, 102.2 ± 11.34 vs 75.25 ± 3.469, P = 0.0528; for area under the response curve (AUC), 7533 ± 763.0 vs 5117 ± 181.4, P<0.05, n = 5 for both). The temporal pattern of the difference was similar to that in neutrophils and T-cells, though the magnitude of the difference in macrophages was smaller than in neutrophils or T-cells, which showed around a 3-fold difference between WT and *Hvcn1^−/−^* cells.^8,14^ Collectively then, ROS production in *Hvcn1^−/−^* animals is enhanced in microglia but reduced in macrophages, suggesting that microglia have a unique mechanism governing ROS production.

### Comparison of ROS production in primary cultured microglia and neutrophils

**Figure 3.**
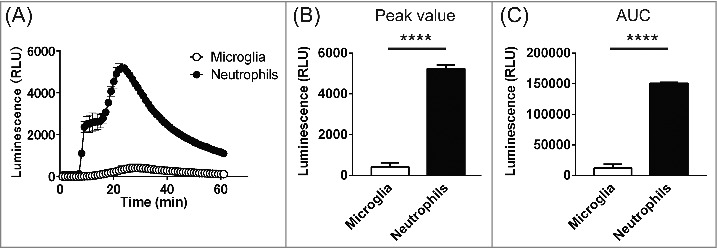
Comparison of ROS production in microglia and neutrophils. (A) Time course of the extracellular superoxide anion signal in microglia (open circles) and neutrophils (filled circles) after stimulation with 1 μM PMA (5 min). The responses correspond to the signal from 1000 cells from both cell types under identical conditions. Data are means ± SEM. P<0.0001, two-way ANOVA, n = 3 for each. (B) Peak luminescent signals in microglia and neutrophils. P<0.0001, unpaired t-test. (C) AUCs calculated by summing the values from the onset of stimulation to the end of recording. P<0.0001, unpaired t-test.

Finally, the levels of ROS production were compared between microglia and neutrophils stimulated using 1μM PMA. Neutrophils exhibited bimodal responses to PMA stimulation (Fig. 3A). Moreover, ROS production by neutrophils was much greater than by microglia (P<0.0001, two-way ANOVA, n = 3 for each), with neutrophils showing about 12-fold greater peak ROS production (426.0 ± 197.6 vs 5,240 ± 170.1; n = 3, P<0.0001) and AUC (12,078 ± 6,059, n = 3 vs 150,427 ± 1,623; n = 3, P<0.0001).

## Discussion

### Activation does not account for the enhanced ROS production in Hvcn1^−/−^ microglia

In the present study, we compared the expression of *gp91* between WT and *Hvcn1*^−/−^ microglia to test the idea that the enhanced ROS production in *Hvcn1*^−/−^ microglia is attributable to a difference in the expression of NADPH oxidase subunits. However, we observed no obvious difference between the two genotypes in that regard. In addition, when we applied LPS, which is known to activate microglia by inducing a pro-inflammatory response,^26^ similar levels of *Tnfa* expression, a widely used inflammatory marker, were detected in WT and *Hvcn1*^−/−^ microglia. Thus, LPS strongly induces activation in both WT and *Hvcn1*^−/−^ microglia. In the present study, microglial activation upregulated *gp91* expression (Fig. 1C), as previously reported.^24,25^ However, there was no obvious difference in *gp91* expression between WT and *Hvcn1*^−/−^ microglia in LPS-treated cells (Fig. 1C). This is consistent with our recent immunohistochemical results showing that expression of p67, a cytosolic NADPH oxidase subunit, is upregulated by microglial activation and that expression of p67 is similar in WT and *Hvcn1*^−/−^ microglia treated with LPS.^14^ On the other hand, enhanced ROS production in *Hvcn1*^−/−^ microglia was still observed in LPS-treated cells. Furthermore, it is noteworthy that ROS production was even higher in vehicle-treated *Hvcn1*^−/−^ microglia than LPS-treated WT microglia (Fig. 1D), though expression of gp91 is relatively lower in vehicle-treated *Hvcn1*^−/−^ microglia than LPS-treated WT microglia (Fig. 1C). These results indicate that expression levels of NADPH oxidase subunits cannot account for the enhanced ROS production in *Hvcn1*^−/−^ microglia.

In addition, while microglial activation by LPS increased ROS production in parallel with shortening of time to peak (Fig. 1D–F), the enhancement in ROS production seen in *Hvcn1*^−/−^ microglia in the absence of LPS was not accompanied by shortening of time to peak (Fig. 1D and our recent study^14^). These observations indicate that the activation status of microglia also does not account for the enhanced ROS production in *Hvcn1*^−/−^ microglia.

### Enhanced ROS production related to Hvcn1 deficiency is unique to microglia

We found that the effect of *Hvcn1* deficiency in macrophages was opposite that of microglia – i.e., ROS production was lower in *Hvcn1*^−/-^ than WT macrophages, though the difference was more moderate than in neutrophils or T-cells. Thus, microglia have a unique mechanism for ROS production that is differently regulated by VSOP/Hv1 than in other cell types. It is noteworthy in that regard that the bimodal response observed in macrophages is common to neutrophils and T-cells, but is not observed in microglia. As shown in Fig. 1D and our recent study,^14^ microglia exhibit a single-peaked response to both PMA and zymosan. When we focused on the bimodal responses observed in macrophages, neutrophils and T-cells, the suppression of ROS production caused by *Hvcn1* deficiency was only observed in the second phase (second peak in the present study; Fig. 2 and our other studies^8,14^). While the mechanism underlying these separate responses is not completely understood, some studies suggest that the two responses are triggered by separate signaling cascades in, perhaps, adhesion-dependent or -independent phases.^27-30^ It is therefore possible that the supportive function of VSOP/Hv1 in ROS production is limited to specific conditions, and microglia lack that supportive function during respiratory bursts.

### Insight into the difference in ROS production between microglia and neutrophils

VSOP/Hv1 supports NADPH oxidase activity by exporting excess protons generated during ROS production. In our recent study,^14^ we suggested the level of ROS production in microglia may be too small to affect membrane potential of these cells,^31^ reducing the need for charge compensation. We based that idea on our observation that PMA did not affect the membrane potential in either WT or *Hvcn1*^−/−^ microglia (Fig S4B in our recent study^14^). By contrast, PMA induces strong depolarization in neutrophils.^6,14^ In the present study, therefore, we quantitatively compared extracellular superoxide anion production between microglia and neutrophils (Fig. 3). As expected, both the peak and integrated (AUC) luminescence signals were an order of magnitude greater with neutrophils than microglia (Fig. 3B and C). Considering the smaller size of neutrophils, it may make sense that microglia do not show as prominent depolarization during respiratory bursts. Thus, the requirement for charge compensation in microglia should not be as high as in neutrophils during respiratory bursts. Nonetheless, in peritoneal macrophages, whose ROS production was also much lower than in neutrophils, ROS production was reduced in *Hvcn1*^−/−^ cells, though the difference was moderate compared to neutrophils (Fig. 2). It thus appears that the charge compensation function of VSOP/Hv1 during respiratory bursting plays some role in macrophages. It will be also important to consider the possibility that other charge compensatory mechanisms suggested by other studies (voltage-gated K^+^ channels, Ca^2+^-activated K^+^ channels and Cl^−^ channels)^32-36^ function during respiratory burst, reducing the necessity of VSOP/Hv1 for charge compensation.

Overall, the present work and our recent study^14^ shed light on the unexpected function of VSOP/Hv1 in microglial ROS regulation. In the future, it will be important to determine the molecular mechanism underlying this unconventional regulation of ROS production in microglia as well as its relationship with neuropathology, which has been suggested by studies of mice deficient in VSOP/Hv1.^12,14,15^

## Materials and Methods

### Reagents

Phorbol myristate acetate (PMA) and lipopolysaccharide (LPS) were purchased from Sigma (St Louis, MO). PMA was dissolved in DMSO at 1.6 mM, and LPS was dissolved in H_2_O at 10 mg/mL. They were kept in −30 ºC.

### Animals

The *Hvcn1*^−/−^ mice used in this study were previously described in detail.^14^ In these animals, exon 5 of *Hvcn1* is flanked by two loxP sites and is completely deleted from the entire body by crossing with CAG-cre transgenic mice.^37^ The elimination of VSOP/Hv1 activity in these mice was confirmed in our earlier study.^14^ All animal procedures were approved by the Animal Care and the Use Committee of Osaka University and Niigata University.

### Preparation of primary microglia

Primary microglia were prepared as described previously.^14^ Briefly, minced cerebral cortices from newborn mice were treated with trypsin and DNaseI. Dissociated and filtered cells were suspended in Dulbecco's modified Eagle medium supplemented with 0.1% penicillin/streptomycin and 10% heat-inactivated fetal bovine serum and then seeded into poly-L-lysine-coated 75 cm^2^ cell culture flasks. The medium was changed appropriately. The floating microglia were collected by gently shaking the flask.

### Measurement of extracellular ROS production by microglia, neutrophils and peritoneal macrophages

Extracellular ROS production by microglia and neutrophils was measured as described previously.^14^ Cells were suspended in balanced salt solution containing (in mM) NaCl 150, KCl 5.0, CaCl_2_ 1.8, MgCl_2_ 1.2, HEPES 25, and D-glucose 10 (pH 7.4). Superoxide anion was monitored using a luminol-based assay, Diogenes (National Diagnostics, Atlanta, GA). The signal was detected as luminescence using a SH-9000Lab microplate reader (Corona Electric, Ibaraki, Japan). For microglia, data were normalized to the number of microglial cells; for neutrophils, all experiments were performed with 1000 cells. For measurement of extracellular ROS production by macrophages, peritoneal macrophages were harvested after injecting ice-cold phosphate buffered saline into the peritoneum and then collecting it.^38^ We calculated the superoxide anion production by subtracting the data obtained after treatment with 100 U/mL superoxide dismutase from the original data.

### Real-time quantitative reverse transcription PCR with cultured microglia

After total RNA was prepared from cultured microglia using TRIzol (Invitrogen Carlsbad, CA) according to the manufacturer's protocol, cDNA was synthesized using a SuperScript III First-Strand Synthesis System (Invitrogen Carlsbad, CA). Gene expression levels were analyzed using the following gene-specific primers: for *gp91*, 5′-CCAACTGGGATAACGAGTTCA-3′ (SE) and 5′-GAGAGTTTCAGCCAAGGCTTC-3′ (AS); for *Tnfa*, 5′-CCACCACGCTCTTCTGTCTAC-3′ (SE) and 5′- GGCTACAGGCTTGTCACTCG-3′ (AS); for *Gapdh*, 5′-TCCTACCCCCAATGTGTCC-3′ (SE) and 5′-ACCTGGTCCTCAGTGTAGCC-3′ (AS). Real-time quantitative reverse transcription PCR was performed using KOD SYBR qPCR Mix (TOYOBO, Tokyo, Japan) and ABI PRISM 7900HT (Applied Biosystems, Foster City, CA). The gene expression level of interest was normalized to that of *Gapdh*.

### Statistical analyses

Statistical analyses were performed using Prism 6 (GraphPad Software, San Diego, CA). All the data are represented as means ± SEM.

## References

[1] Sasaki M, Takagi M, Okamura Y A voltage sensor-domain protein is a voltage-gated proton channel. Science. 2006;312:589-92. doi:10.1126/science.1122352. PMID:16556803. 16556803

[2] Ramsey IS, Moran MM, Chong JA, Clapham DE A voltage-gated proton-selective channel lacking the pore domain. Nature. 2006;440:1213-16. doi:10.1038/nature04700. PMID:16554753. 16554753 PMC4084761

[3] DeCoursey TE. Voltage-gated proton channels: molecular biology, physiology, and pathophysiology of the H(V) family. Physiol Rev. 2013;93:599-652. doi:10.1152/physrev.00011.2012. PMID:23589829. 23589829 PMC3677779

[4] Ramsey IS, Ruchti E, Kaczmarek JS, Clapham DE Hv1 proton channels are required for high-level NADPH oxidase-dependent superoxide production during the phagocyte respiratory burst. Proc Natl Acad Sci U S A. 2009;106:7642-7. doi:10.1073/pnas.0902761106. PMID:19372380. 19372380 PMC2669790

[5] Okochi Y, Sasaki M, Iwasaki H, Okamura Y Voltage-gated proton channel is expressed on phagosomes. Biochem Biophys Res Commun. 2009;382:274-9. doi:10.1016/j.bbrc.2009.03.036. PMID:19285483. 19285483

[6] El Chemaly A, Okochi Y, Sasaki M, Arnaudeau S, Okamura Y, Demaurex N VSOP/Hv1 proton channels sustain calcium entry, neutrophil migration, and superoxide production by limiting cell depolarization and acidification. J Exp Med. 2010;207:129-39. doi:10.1084/jem.20091837. PMID:20026664. 20026664 PMC2812533

[7] Capasso M, Bhamrah MK, Henley T, Boyd RS, Langlais C, Cain K, Dinsdale D, Pulford K, Khan M, Musset B, et al. HVCN1 modulates BCR signal strength via regulation of BCR-dependent generation of reactive oxygen species. Nat Immunol. 2010;11:265-72. doi:10.1038/ni.1843. PMID:20139987. 20139987 PMC3030552

[8] Sasaki M, Tojo A, Okochi Y, Miyawaki N, Kamimura D, Yamaguchi A, Murakami M, Okamura Y Autoimmune disorder phenotypes in Hvcn1-deficient mice. Biochem J. 2013;450:295-301. doi:10.1042/BJ20121188. PMID:23231444. 23231444

[9] Zhu X, Mose E, Zimmermann N Proton channel HVCN1 is required for effector functions of mouse eosinophils. BMC Immunol. 2013;14:24. doi:10.1186/1471-2172-14-24. PMID:23705768. 23705768 PMC3668235

[10] Petheo GL, Orient A, Barath M, Kovacs I, Rethi B, Lanyi A, Rajki A, Rajnavölgyi E, Geiszt M Molecular and functional characterization of Hv1 proton channel in human granulocytes. PloS One. 2010;5:e14081. doi:10.1371/journal.pone.0014081. PMID:21124855. 21124855 PMC2990768

[11] El Chemaly A, Nunes P, Jimaja W, Castelbou C, Demaurex N Hv1 proton channels differentially regulate the pH of neutrophil and macrophage phagosomes by sustaining the production of phagosomal ROS that inhibit the delivery of vacuolar ATPases. J Leukoc Biol. 2014;95:827-39. doi:10.1189/jlb.0513251. PMID:24415791. 24415791

[12] Wu LJ, Wu G, Akhavan Sharif MR, Baker A, Jia Y, Fahey FH, Luo HR, Feener EP, Clapham DE The voltage-gated proton channel Hv1 enhances brain damage from ischemic stroke. Nat Neurosci. 2012;15:565-73. doi:10.1038/nn.3059. PMID:22388960. 22388960 PMC3314139

[13] DeCoursey TE, Morgan D, Cherny VV The voltage dependence of NADPH oxidase reveals why phagocytes need proton channels. Nature. 2003;422:531-4. doi:10.1038/nature01523. PMID:12673252. 12673252

[14] Kawai T, Okochi Y, Ozaki T, Imura Y, Koizumi S, Yamazaki M, Abe M, Sakimura K, Yamashita T, Okamura Y Unconventional role of voltage-gated proton channels (VSOP/Hv1) in regulation of microglial ROS production. J Neurochem. 2017;142:686-99. doi:10.1111/jnc.14106. PMID:28628214. 28628214

[15] Liu J, Tian D, Murugan M, Eyo UB, Dreyfus CF, Wang W, Wu LJ Microglial Hv1 proton channel promotes cuprizone-induced demyelination through oxidative damage. J Neurochem. 2015;135:347-56. doi:10.1111/jnc.13242. PMID:26173779. 26173779 PMC4721248

[16] Wilkinson BL, Landreth GE The microglial NADPH oxidase complex as a source of oxidative stress in Alzheimer's disease. J Neuroinflammation. 2006;3:30. doi:10.1186/1742-2094-3-30. PMID:17094809. 17094809 PMC1637099

[17] Chanock SJ, el Benna J, Smith RM, Babior BM The respiratory burst oxidase. J Biol Chem. 1994;269:24519-22. PMID:7929117. 7929117

[18] Dinauer MC. The respiratory burst oxidase and the molecular genetics of chronic granulomatous disease. Crit Rev Clin Lab Sci. 1993;30:329-69. doi:10.3109/10408369309082591. PMID:8110374. 8110374

[19] Segal AW. How neutrophils kill microbes. Annu Rev Immunol. 2005;23:197-223. doi:10.1146/annurev.immunol.23.021704.115653. PMID:15771570. 15771570 PMC2092448

[20] Chen J, He R, Minshall RD, Dinauer MC, Ye RD Characterization of a mutation in the Phox homology domain of the NADPH oxidase component p40phox identifies a mechanism for negative regulation of superoxide production. J Biol Chem. 2007;282:30273-84. doi:10.1074/jbc.M704416200. PMID:17698849. 17698849

[21] Rasmussen I, Pedersen LH, Byg L, Suzuki K, Sumimoto H, Vilhardt F Effects of F/G-actin ratio and actin turn-over rate on NADPH oxidase activity in microglia. BMC Immunol. 2010;11:44. doi:10.1186/1471-2172-11-44. PMID:20825680. 20825680 PMC2944333

[22] Tamura M, Kai T, Tsunawaki S, Lambeth JD, Kameda K Direct interaction of actin with p47(phox) of neutrophil NADPH oxidase. Biochem Biophys Res Commun. 2000;276:1186-90. doi:10.1006/bbrc.2000.3598. PMID:11027608. 11027608

[23] Zhan Y, He D, Newburger PE, Zhou GW p47(phox) PX domain of NADPH oxidase targets cell membrane via moesin-mediated association with the actin cytoskeleton. J Cell Biochem. 2004;92:795-809. doi:10.1002/jcb.20084. PMID:15211576. 15211576

[24] Qin L, Crews FT NADPH oxidase and reactive oxygen species contribute to alcohol-induced microglial activation and neurodegeneration. J Neuroinflammation. 2012;9:5. doi:10.1186/1742-2094-9-5. PMID:22240163. 22240163 PMC3271961

[25] Dohi K, Ohtaki H, Nakamachi T, Yofu S, Satoh K, Miyamoto K, Song D, Tsunawaki S, Shioda S, Aruga T Gp91phox (NOX2) in classically activated microglia exacerbates traumatic brain injury. J Neuroinflammation. 2010;7:41. doi:10.1186/1742-2094-7-41. PMID:20659322. 20659322 PMC2917406

[26] Nakamura Y, Si QS, Kataoka K Lipopolysaccharide-induced microglial activation in culture: temporal profiles of morphological change and release of cytokines and nitric oxide. Neurosci Res. 1999;35:95-100. doi:10.1016/S0168-0102(99)00071-1. PMID:10616913. 10616913

[27] Nathan CF. Neutrophil activation on biological surfaces. Massive secretion of hydrogen peroxide in response to products of macrophages and lymphocytes. J Clin Invest. 1987;80:1550-60. doi:10.1172/JCI113241. PMID:2445780. 2445780 PMC442423

[28] Berton G, Laudanna C, Sorio C, Rossi F Generation of signals activating neutrophil functions by leukocyte integrins: LFA-1 and gp150/95, but not CR3, are able to stimulate the respiratory burst of human neutrophils. J Cell Biol 1992;116:1007-17. doi:10.1083/jcb.116.4.1007. PMID:1346398. 1346398 PMC2289342

[29] Berton G, Lowell CA Integrin signalling in neutrophils and macrophages. Cell Signal. 1999;11:621-35. doi:10.1016/S0898-6568(99)00003-0. PMID:10530871. 10530871

[30] Fumagalli L, Campa CC, Germena G, Lowell CA, Hirsch E, Berton G Class I phosphoinositide-3-kinases and SRC kinases play a nonredundant role in regulation of adhesion-independent and -dependent neutrophil reactive oxygen species generation. J Immunol. 2013;190:3648-60. doi:10.4049/jimmunol.1201951. PMID:23447687. 23447687 PMC4280093

[31] Nathan C, Shiloh MU Reactive oxygen and nitrogen intermediates in the relationship between mammalian hosts and microbial pathogens. Proc Natl Acad Sci U S A. 2000;97:8841-48. doi:10.1073/pnas.97.16.8841. PMID:10922044. 10922044 PMC34021

[32] Fordyce CB, Jagasia R, Zhu X, Schlichter LC Microglia Kv1.3 channels contribute to their ability to kill neurons. The Journal of neuroscience: the official journal of the Society for Neuroscience. 2005;25:7139-49. doi:10.1523/JNEUROSCI.1251-05.2005. PMID:16079396. 16079396 PMC6725234

[33] De Simoni A Allen NJ, Attwell D Charge compensation for NADPH oxidase activity in microglia in rat brain slices does not involve a proton current. Eur J Neurosci. 2008;28:1146-56. doi:10.1111/j.1460-9568.2008.06417.x. PMID:18783372. 18783372 PMC2628425

[34] Thomas MP, Chartrand K, Reynolds A, Vitvitsky V, Banerjee R, Gendelman HE Ion channel blockade attenuates aggregated alpha synuclein induction of microglial reactive oxygen species: relevance for the pathogenesis of Parkinson's disease. J Neurochem. 2007;100:503-19. doi:10.1111/j.1471-4159.2006.04315.x. PMID:17241161. 17241161

[35] Khanna R, Roy L, Zhu X, Schlichter LC K+ channels and the microglial respiratory burst. Am J Physiol Cell Physiol. 2001;280:C796−806. PMID:11245596. 11245596 10.1152/ajpcell.2001.280.4.C796

[36] Nguyen HM, Blomster LV, Christophersen P, Wulff H Potassium channel expression and function in microglia: Plasticity and possible species variations. Channels (Austin). 2017;11:305-15. doi:10.1080/19336950.2017.1300738. PMID:28277939. 28277939 PMC5555259

[37] Sakai K, Miyazaki J A transgenic mouse line that retains Cre recombinase activity in mature oocytes irrespective of the cre transgene transmission. Biochem Biophys Res Commun. 1997;237:318-24. doi:10.1006/bbrc.1997.7111. PMID:9268708. 9268708

[38] Zhang X, Goncalves R, Mosser DM The isolation and characterization of murine macrophages. Current protocols in immunology. 2008; 83:14.1.1-14.1.14. doi:10.1002/0471142735.im1401s83. PMID:19016445. 19016445 PMC2834554

